# Cribriform pattern and IDC‐P in prostate biopsies: prognostic relevance and reporting in metastatic disease

**DOI:** 10.1002/2056-4538.70052

**Published:** 2025-10-08

**Authors:** Yoichiro Okubo, Rika Kasajima, Shinya Sato, Yayoi Yamamoto, Atsuto Suzuki, Tomohiko Aigase, Shu Yuguchi, Chie Hasegawa, Emi Yoshioka, Kota Washimi, Ryotaro Matsuyama, Yukihiko Hiroshima, Noboru Nakaigawa, Hiroto Narimatsu, Takeshi Kishida, Tomoyuki Yokose, Yohei Miyagi

**Affiliations:** ^1^ Department of Pathology Kanagawa Cancer Center Yokohama Japan; ^2^ Molecular Pathology and Genetics Division Kanagawa Cancer Center Research Institute Yokohama Japan; ^3^ Department of Radiology Kanagawa Cancer Center Yokohama Japan; ^4^ Department of Urology Kanagawa Cancer Center Yokohama Japan; ^5^ Department of Cancer Genome Medicine Kanagawa Cancer Center Yokohama Japan; ^6^ Advanced Cancer Therapeutics Division Kanagawa Cancer Center Research Institute Yokohama Japan; ^7^ Department of Genetic Medicine Kanagawa Cancer Center Yokohama Japan; ^8^ Cancer Prevention and Cancer Control Division Kanagawa Cancer Center Research Institute Yokohama Japan; ^9^ Department of Pathology Odawara Municipal Hospital Odawara‐shi Japan

**Keywords:** prostate carcinoma, cribriform pattern, intraductal carcinoma of the prostate, biopsy, metastasis

## Abstract

Cribriform pattern and intraductal carcinoma of the prostate are recognized adverse histological features, yet their prognostic value in treatment‐naïve metastatic disease remains uncertain. We conducted a single‐center retrospective study of 183 biopsy‐proven prostate carcinomas (105 with metastatic castration‐sensitive prostate carcinoma and 78 non‐metastatic high‐grade cases) diagnosed between 2017 and 2024. Cribriform pattern, intraductal carcinoma of the prostate, and coagulative tumor necrosis were recorded per core and summarized as patient‐level binary status and as semiquantitative proportions per cancer‐positive core. Two multivariable logistic regression models (binary and semiquantitative) were fitted, and receiver operating characteristic (ROC) analysis evaluated the discriminatory performance of the cribriform proportion. Cribriform pattern and intraductal carcinoma of the prostate were more frequent in metastatic castration‐sensitive prostate carcinoma. In the semiquantitative model, the cribriform proportion remained independently associated with metastatic status [odds ratio (OR) 1.29, 95% CI 1.07–1.55, *p* = 0.008; per 1.0 increase in the proportion, equivalent to OR 1.03 per 10%‐point increase], whereas necrosis remained significant only in the binary model. The cancer‐positive core rate and a lower total number of biopsy cores were predictive in both models, whereas prostate‐specific antigen, intraductal carcinoma of the prostate, and Grade Group composition were not independent predictors. ROC analysis for the cribriform proportion yielded an area under the curve of 0.704, with a Youden Index cut‐off of 0.445 (approximately half of cancer‐positive cores), corresponding to a sensitivity of 57.1% and a specificity of 75.6%. These findings indicate that semiquantitative reporting of cribriform pattern – expressed as the proportion of cancer‐positive cores – adds discriminatory information for metastatic status at presentation and could complement binary reporting in high‐grade disease. From a clinical perspective, such evaluation may refine risk stratification at diagnosis and support treatment intensification strategies in very‐high‐risk patients.

## Introduction

Accurate histological assessment underpins risk stratification in prostate carcinoma, particularly in high‐grade or advanced disease. Although the Grade Group (GG) system remains the mainstay of grading, additional architectural features contribute independent prognostic information [1]. Among these, cribriform pattern and intraductal carcinoma of the prostate (IDC‐P) have emerged as adverse markers not fully captured by GG alone [2, 3]. Previously, we reported that the binary presence of cribriform pattern or IDC‐P in biopsy was associated with lymph‐node metastasis at prostatectomy, that is, an N‐stage endpoint [4]. Clinically, metastatic castration‐sensitive prostate carcinoma (mCSPC) is often diagnosed on initial biopsy with treatment‐naïve primary tissue available, providing an opportunity to interrogate histopathology at presentation – here focusing on metastatic status at diagnosis (M‐stage) [5]. Given consensus definitions for cribriform morphology [6], we hypothesized that a semiquantitative approach – proportion of cancer‐positive cores showing cribriform pattern – might outperform binary scoring for distinguishing metastatic from non‐metastatic status at diagnosis, consistent with contemporary summaries of biopsy practice [7].

## Materials and methods

### Case selection

We queried our institutional pathology database to identify all patients with biopsy‐proven prostatic adenocarcinoma between 2017 and 2024. We included all treatment‐naïve patients who had mCSPC at presentation, defined as M1 disease on the initial staging work‐up (contrast‐enhanced computed tomography (CT) and whole‐body bone scintigraphy; prostate magnetic resonance imaging (MRI) was routinely performed) before any systemic therapy. Prostate‐specific membrane antigen positron emission tomography/CT (PSMA PET/CT) was not uniformly available across the study period and was not used to define metastatic status, which may have led to under‐detection of metastatic disease. As the non‐mCSPC comparator, we intentionally selected GG 5 cases without regional nodal or distant metastasis at diagnosis, to match the highest‐grade histology typically encountered in mCSPC and minimize confounding by grade heterogeneity. These non‐metastatic cases are hereafter referred to as non‐metastatic castration‐sensitive prostate carcinoma (non‐mCSPC). Non‐metastatic status was determined at the time of diagnosis based on the initial staging work‐up; subsequent development of metastasis during follow‐up was outside the scope of this study. We chose a GG 5 comparator rather than Gleason‐score matching because the mCSPC cohort spans Gleason 7–10 and is enriched for high‐grade disease; restricting the comparator to GG 5 allowed us to test whether the semiquantitative cribriform burden adds discriminative value within high‐grade disease [1]. We excluded cases with prior therapy, small‐cell/neuroendocrine differentiation, or inadequate material [8]. All sampling‐intensity metrics (i.e., the total number of biopsy cores per patient) reflected routine clinical practice and were abstracted from pathology reports; all biopsy cores obtained per patient were reviewed and included in the analysis, and no analytic subsampling of cores within a case was performed. Sampling intensity (the total number of biopsy cores per patient) was included as a continuous covariate in multivariable models to account for its potential confounding effect. Information on whether biopsies were performed under MRI/ultrasound guidance was not systematically captured during the study period; accordingly, guidance modality was not compared between groups or included in the analysis. This limitation precluded formal comparison between groups and may have introduced detection bias, which is addressed in the Limitations section – notably, MRI‐abnormality‐targeted sampling can raise ≥GG2 detection and upgrade highest GG versus systematic biopsy [9].

### Histological evaluation

**Figure 1:**
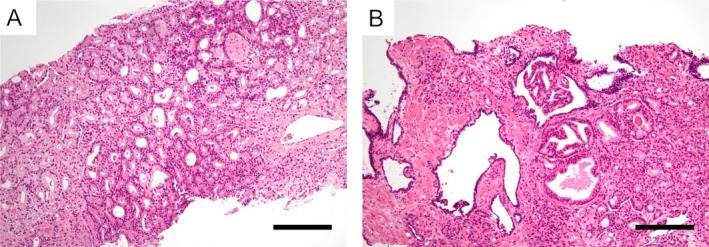
Representative histological features of the cribriform pattern and intraductal carcinoma of the prostate (IDC‐P). (A) Cribriform pattern, characterized by a confluent proliferation of malignant epithelial cells forming multiple rounded glandular lumina, often referred to as ‘punched‐out’ spaces. These lumina are easily recognized at low magnification and lack intervening fibrovascular stroma or capillaries. This architecture corresponds to the Gleason pattern 4 morphology. (B) IDC‐P demonstrating dense epithelial proliferation confined within pre‐existing glandular ducts that retain their basal contours. Although morphologically similar to the cribriform pattern, the lesion remains enclosed within native duct structures, with apparent preservation of the basal cell layer. Both panels show hematoxylin and eosin‐stained prostate needle biopsy specimens at ×100 magnification. Scale bars represent 200 μm.

Clinicopathological parameters – including age, prostate‐specific antigen (PSA) level, body mass index (BMI), highest GG, GG composition, total number of biopsy cores, and the number of cancer‐positive cores – were extracted from the institutional pathology database. The cribriform pattern was defined in accordance with the International Society of Urological Pathology (ISUP) consensus [6]. For reporting clarity, we additionally used a size descriptor. ‘Large cribriform’ was defined as a cribriform structure whose maximal diameter was at least twice that of the largest adjacent benign gland. Conversely, smaller lesions were termed ‘small’, consistent with previous reports [10]. If both large and small lesions were present in a patient, the case was classified as large for patient‐level analyses. This size descriptor was used in supplementary analyses; our prespecified primary endpoint remained the semiquantitative per‐core cribriform proportion. With hematoxylin and eosin (H&E) staining, invasive cribriform carcinoma was diagnosed when a confluent epithelial proliferation with multiple rounded lumina extended beyond pre‐existing duct–acinar confines, typically showing an irregular/infiltrative outer border, loss of basal contours, desmoplastic stromal response, and/or extraluminal comedo‐type necrosis [6]. In contrast, IDC‐P was diagnosed when cribriform, solid, or micropapillary proliferation remained confined within markedly expanded native ducts or acini with sharply preserved basal contours (i.e., a peripheral ‘two‐cell’ appearance) and intact periductal stroma; central necrosis and bridging were permitted provided confinement was maintained [11]. Consistent with ISUP recommendations, basal‐cell immunostains were not routinely performed. All H&E‐stained biopsy slides were independently reviewed by three board‐certified pathologists (YO, SS, and YM), consistent with evidence that expert re‐review at high‐volume centers improves biopsy–prostatectomy GG concordance [12]. Patient‐level (‘binary’) status for each feature (cribriform pattern, IDC‐P, and coagulative tumor necrosis) was assigned at a consensus review; when disagreement persisted, the final arbitration was provided by the most senior pathologist (YM), consistent with our previous approach [4]. All biopsy cores obtained per patient were reviewed and included; cases with missing or technically inadequate core slides were classified as inadequate and excluded according to prespecified criteria. For semiquantitative assessments, the denominator was the number of cancer‐positive cores in each case. The GG 4 + 5 composition ratio was defined as the proportion of cancer‐positive cores assigned to GG 4 or GG 5. Cases that remained indeterminate after review were designated not applicable (N/A) and excluded from multivariable modelling and ROC analyses. For clarity, the GG 4 + 5 composition ratio was calculated as the proportion of cancer‐positive biopsy cores assigned to GG 4 or 5, and the GG 5 composition ratio as the proportion of cancer‐positive cores assigned to GG 5. The denominator in both cases was the number of cancer‐positive cores per patient. Representative histological features of the cribriform pattern and IDC‐P are illustrated in Figure 1.

### Statistical analysis

Continuous variables were tested for normality using the Shapiro–Wilk test. Depending on the distribution, group comparisons were performed using either Student's *t*‐test or the Mann–Whitney *U* test for continuous and semiquantitative variables and Fisher's exact test for binary categorical variables. For multivariable analysis, logistic regression models were used to evaluate predictors of metastatic status. Two modelling approaches were used: (1) a binary model, based on the presence or absence of each histological feature at the patient level; and (2) a semiquantitative model, incorporating proportional metrics per cancer‐positive core. Variables were selected for inclusion in multivariable models based on univariable significance (*p* < 0.10) and *a priori* clinical relevance; sensitivity analyses including key clinical covariates were performed to confirm robustness of findings. Standardized regression coefficients (*β*), scaled to one standard deviation of the predictor, were calculated to assess relative contribution. Receiver operating characteristic (ROC) curve analysis was additionally performed to evaluate the discriminatory power of the cribriform pattern proportion in predicting metastatic status. The area under the curve (AUC) was calculated, and the optimal cut‐off value was determined using the Youden Index, defined as the point maximizing the sum of sensitivity and specificity minus one [13]. The resulting threshold was further considered in relation to clinical interpretability. All statistical analyses were conducted using IBM SPSS Statistics, version 29.0 (IBM Corp., Armonk, NY, USA). A *p* value <0.05 was considered statistically significant. Guidance modality (MRI/ultrasound targeting) was unavailable and therefore not included as a covariate. Size‐stratified analyses (none, small, large) were prespecified as descriptive and were not included in multivariable models to avoid collinearity with the semiquantitative per‐core cribriform proportion and to preserve model parsimony. In an exploratory, post hoc composite assessment, we summarized the cribriform proportion with binary tumor necrosis into three patient‐level categories (neither, either alone, both) and presented descriptive cross‐tabulation and univariable odds ratios (ORs); composite variables were not entered into multivariable models to avoid collinearity and preserve model parsimony. ORs and 95% confidence intervals (CI) were reported.

### Ethical considerations

This study was conducted in accordance with the principles of the Declaration of Helsinki and was approved by the Ethics Review Committee of Kanagawa Cancer Center (approval number: 2019‐eki‐36; renewed: 11 February 2025). Written informed consent was obtained from all patients for their participation in this research and for the publication of the study data.

## Results

Baseline clinicopathological characteristics of the two cohorts are summarized in Table 1. A total of 183 patients were included, comprising 105 patients with mCSPC and 78 patients with non‐mCSPC. The median age was 75 years in both groups (range: 47–88 and 52–88 years, respectively; *p* = 0.309, Mann–Whitney *U* test). In the mCSPC cohort, the highest biopsy Gleason score ranged from 7 to 10. Serum PSA levels were significantly higher in the mCSPC group (median: 183.5 ng/ml; range: 1.7–20,000) than in the non‐mCSPC group (median: 14.75 ng/ml; range: 4.1–412; *p* < 0.001, Mann–Whitney *U* test). BMI was comparable between the groups (mCSPC: median, 23.22; range: 14.93–31.19 versus non‐mCSPC: median, 23.23; range: 16.34–36.15; *p* = 0.748, Mann–Whitney *U* test). The total number of biopsy cores was significantly lower in the mCSPC group (median: 10; range: 2–20) than in the non‐mCSPC group (median: 12; range: 4–14; *p* < 0.001, Mann–Whitney *U* test). This variable was included as a covariate in multivariable models. The cancer‐positive core ratio was significantly higher in the mCSPC group (median: 0.90; range: 0.18–1.00) than in the non‐mCSPC group (median: 0.58; range: 0.13–1.00; *p* < 0.001, Mann–Whitney *U* test). The total number of cancer‐positive cores did not differ significantly between groups (mCSPC: median, 6.00; range: 1.00–14.00 versus non‐mCSPC: median, 6.50; range: 2.00–13.00; *p* = 0.062, Mann–Whitney *U* test).

**Table 1:** Distribution and univariate comparison of clinicopathological parameters between patients with mCSPC and non‐mCSPC

Parameter	mCSPC (*n* = 105)	Non‐mCSPC (*n* = 78)	*p*
Age (years, median)	75; 47–88	75; 52–88	0.309
PSA (ng/ml, median; range)	183.5; 1.7–20,000	14.75; 4.1–412	<0.001
BMI (median)	23.22 (14.93–31.19)	23.23 (16.34–36.15)	0.748
Total biopsy cores (median; range)	10; 2–20	12; 4–14	<0.001
Total number of cancer‐positive cores	6.00; 1.00–14.00	6.50; 2.00–13.00	0.062
Cancer‐positive core ratio (median; range)	0.90; 0.18–1.00	0.58; 0.13–1.00	<0.001
Cribriform pattern (binary)	82/105 (78.1%)	46/78 (59.0%)	<0.001
Cribriform pattern (per cancer‐positive core)	0.50; 0.00–1.00	0.18; 0.00–1.00	<0.001
IDC‐P (binary)	72/105 (68.6%)	30/78 (38.5%)	<0.001
IDC‐P (per cancer‐positive core)	0.25; 0.00–1.00	0.00; 0.00–0.67	<0.001
Necrosis (binary)	34/105 (32.4%)	9/78 (11.5%)	<0.001
Necrosis (per cancer‐positive core)	0.00; 0.00–1.00	0.00; 0.00–0.92	0.002
GG 4 + 5 composition ratio	1.00; 0.00–1.00	0.80; 0.20–1.00	<0.001
GG 5 composition ratio	0.62; 0.00–1.00	0.40; 0.00–1.00	<0.001

This table summarizes the distribution and univariate comparison of clinicopathological parameters between patients with mCSPC and non‐mCSPC. Continuous variables are represented as medians with ranges; binary variables are reported as patient counts and percentages. The *p* values were calculated using the Mann–Whitney *U* test for continuous and semiquantitative variables and Fisher's exact test for binary categorical variables. A *p* value of <0.05 was considered statistically significant. Both binary and semiquantitative evaluations are provided for cribriform pattern, IDC‐P, and necrosis. Significant differences were observed in PSA levels, cancer‐positive core ratio, total biopsy cores, cribriform pattern (binary and semiquantitative), IDC‐P (binary and semiquantitative), tumor necrosis (binary and semiquantitative), and GG composition ratios.

BMI, body mass index; GG, Grade Group; IDC‐P, intraductal carcinoma of the prostate; mCSPC, metastatic castration‐sensitive prostate carcinoma; non‐mCSPC, non‐metastatic castration‐sensitive prostate carcinoma; PSA, prostate‐specific antigen.

In a patient‐level descriptive analysis stratified by cribriform size (none, small, large), distributions differed between cohorts (Table 2). Large cribriform was observed more frequently in mCSPC than in non‐mCSPC (66/105, 62.9% versus 32/78, 41.0%), whereas small cribriform was less frequent (11/105, 10.5% versus 18/78, 23.1%). The overall association across categories was statistically significant (Pearson's *χ* ^2^ = 9.71, df = 2, *p* = 0.008), and a linear‐by‐linear trend was present (*χ* ^2^ = 5.44, *p* = 0.020). The cribriform pattern was more frequent in the mCSPC group than in the non‐mCSPC group in both binary evaluation (82 of 105, 78.1% versus 46 of 78 patients, 59.0%; *p* < 0.001, Fisher's exact test) and semiquantitative evaluation (median: 0.50; range: 0.00–1.00 versus median: 0.18; range: 0.00–1.00; *p* < 0.001, Mann–Whitney *U* test). IDC‐P was more frequently observed in the mCSPC group in both binary evaluation (72 of 105 patients, 68.6% versus 30 of 78 patients, 38.5%; *p* < 0.001, Fisher's exact test) and semiquantitative evaluation (median: 0.25; range: 0.00–1.00 versus median: 0.00; range: 0.00–0.67; *p* < 0.001, Mann–Whitney *U* test), but it did not remain an independent predictor in multivariable models. Tumor necrosis was more frequent in the mCSPC group in both binary evaluation (34 of 105 patients, 32.4% versus 9 of 78 patients, 11.5%; *p* < 0.001, Fisher's exact test) and semiquantitative evaluation (median: 0.00; range: 0.00–1.00 versus median: 0.00; range: 0.00–0.92; *p* = 0.002, Mann–Whitney *U* test). The GG 4 + 5 composition ratio was significantly higher in the mCSPC group (median: 1.00; range: 0.00–1.00) than in the non‐mCSPC group (median: 0.80; range: 0.20–1.00; *p* < 0.001, Mann–Whitney *U* test). The GG 5 composition ratio was significantly higher in the mCSPC group (median: 0.62; range: 0.00–1.00) than in the non‐mCSPC group (median: 0.40; range: 0.00–1.00; *p* < 0.001, Mann–Whitney *U* test). These clinicopathological characteristics are summarized in Table 1. In an exploratory composite analysis of the cribriform proportion with binary tumor necrosis (neither, either alone, both), metastatic frequency increased stepwise across categories (38.5%, 60.6%, 75.7%). Univariable logistic regression showed higher odds versus ‘neither’ for ‘either’ (OR 2.47, 95% CI 1.23–4.94, *p* = 0.011) and ‘both’ (OR 4.98, 95% CI 1.95–12.7, *p* < 0.001). These composite variables were not entered into multivariable models. These data are provided in supplementary material, Table S1.

**Table 2:** Patient‐level distribution of cribriform morphology by size (none, small, large) stratified by metastatic status at diagnosis

Cribriform morphology	Non‐mCSPC (*n* = 78)	mCSPC (*n* = 105)	Total (*n* = 183)
None	28 (50.0%)	28 (50.0%)	56
Small	18 (62.1%)	11 (37.9%)	29
Large	32 (32.7%)	66 (67.3%)	98
Total	78 (42.6%)	105 (57.4%)	183

Values are *n* (row %) unless stated. Large cribriform was defined as a cribriform structure whose maximal diameter is at least twice that of the largest adjacent benign gland; lesions below this threshold were termed small. Classification was patient‐level: if both large and small lesions were present, patients were classified as large; otherwise small; otherwise none. Pearson's *χ* ^2^ tested independence across categories; linear‐by‐linear association tested for trend across ordered groups. The *p* values are two‐sided. Percentages may not total 100% due to rounding.

mCSPC, metastatic castration‐sensitive prostate carcinoma; non‐mCSPC, non‐metastatic castration‐sensitive prostate carcinoma.

Multivariable logistic regression analysis was conducted using both binary and semiquantitative models to identify independent predictors of metastatic status. In the binary model, the cancer‐positive core ratio (*p* = 0.050), tumor necrosis (*p* = 0.025), and the number of biopsy cores (OR 0.95; 95% CI 0.92–0.96; *p* < 0.001) remained significant. In the semiquantitative model, the cribriform pattern proportion (OR 1.29; 95% CI 1.07–1.55; *p* = 0.008; per 1.0 increase in the proportion, equivalent to OR 1.03 per 10%‐point increase), the cancer‐positive core ratio (OR 1.42; 95% CI 1.09–1.83; *p* = 0.009), and the number of biopsy cores (OR 0.95; 95% CI 0.93–0.97; *p* < 0.001) were independent predictors. These results are summarized in Table 3.

**Table 3:** Multivariable logistic regression analysis for predictors of metastatic status

Parameter	Binary model (patient level)					Semiquantitative model (per core)		
	95% CI (*β*)	*p*	OR	95% CI (OR)	95% CI (*β*)	*p*	OR	95% CI (OR)
Cancer‐positive core rate	0.000–0.555	0.050	1.16	1.0–1.74	0.090–0.609	0.009	1.42	1.09–1.83
PSA	Narrow range	0.291	1.07	Narrow range	Narrow range	0.285	1.07	Narrow range
Cribriform pattern	−0.072 to 0.213	0.336	1.07	0.93–1.24	0.067–0.435	0.008	1.29	1.07–1.55
IDC‐P	−0.055 to 0.228	0.223	1.09	0.95–1.26	−0.123 to 0.472	0.248	1.09	0.88–1.6
Necrosis	0.021–0.315	0.025	1.15	1.02–1.37	−0.298 to 0.310	0.967	1.0	0.74–1.36
Number of biopsy cores	−0.078 to −0.041	<0.001	0.95	0.92–0.96	−0.070 to −0.031	<0.001	0.95	0.93–0.97
GG 4 + 5 composition ratio (%)	−0.226 to 0.328	0.717	1.02	0.8–1.39	−0.252 to 0.302	0.859	1.01	0.78–1.35

This table summarizes the results of multivariate logistic regression analysis for predictors of metastatic status. Each parameter represents either the presence or the proportional burden of a specific histological feature assessed on biopsy specimens. Binary model values indicate classification based on the presence or absence of each feature at the patient level, while semiquantitative model values reflect the proportion of cancer‐positive cores exhibiting the feature. The odds ratio and 95% confidence interval were derived from the corresponding regression coefficients. All variables were included in the multivariate logistic regression analysis. Statistically significant predictors (*p* < 0.05) are highlighted in the main text. For prostate‐specific antigen (PSA), the confidence interval is described as a narrow range. Due to the extremely small coefficient, SPSS displayed the interval as ‘0.000–0.000’, which reflects the minimal scale of effect and does not imply an actual value of zero. For proportional variables (e.g., cribriform pattern per cancer‐positive core, cancer‐positive core rate), values were scaled 0–1. Odds ratios are expressed per 1.0 increase (i.e., 100‐percentage‐point increase). For interpretability, an approximate odds ratio per 10‐percentage‐point increase can be calculated as exp(*β* × 0.1).

CI, confidence interval; OR, odds ratio; PSA, prostate‐specific antigen.

**Figure 2:**
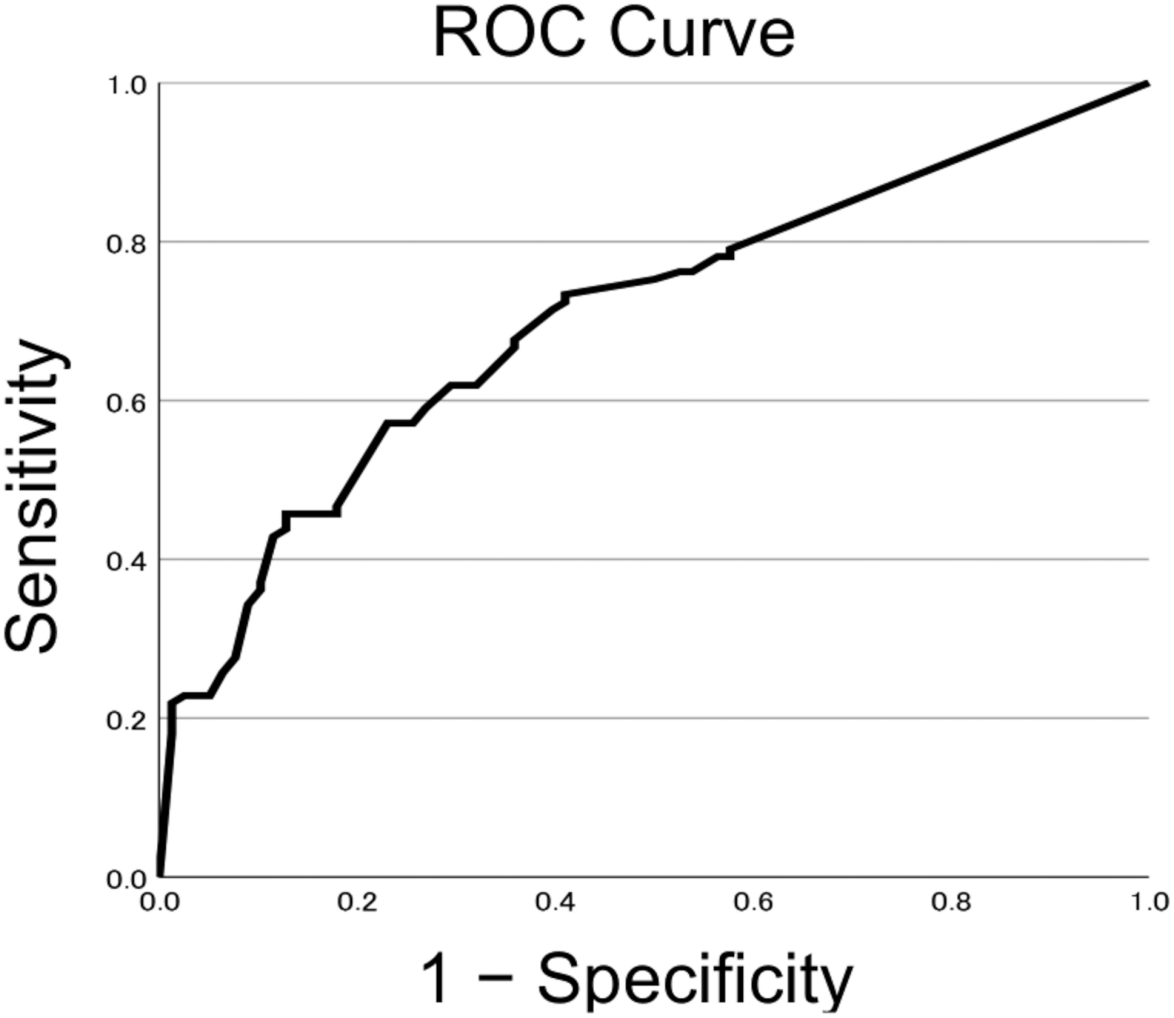
Receiver operating characteristic (ROC) curve evaluating the predictive value of cribriform pattern distribution for metastatic status. The curve was generated based on the proportion of biopsy cores positive for cancer that exhibited a cribriform pattern. The area under the curve (AUC) was 0.704, indicating moderate discriminative accuracy. The optimal cut‐off value, determined using the Youden Index, was 0.445, corresponding to a sensitivity of 57.1% and a specificity of 75.6% The *x*‐axis and *y*‐axis represent 1 − specificity and sensitivity, respectively.

ROC curve analysis was performed to evaluate the discriminatory ability of the cribriform pattern proportion for predicting metastatic status. The AUC was 0.704. The optimal cut‐off value, determined using the Youden Index [13], was 0.445, corresponding to a sensitivity of 57.1% and a specificity of 75.6%. The ROC curve is shown in Figure 2, and detailed parameters are summarized in Table 4.

**Table 4:** Receiver operating characteristic (ROC) curve analysis of the proportion of cancer‐positive cores showing a cribriform pattern in predicting metastatic status

Parameter	AUC	Optimal cut‐off	Sensitivity	Specificity
Cribriform pattern per cancer‐positive core	0.704	0.445	0.571	0.756

This table summarizes the ROC curve analysis for predicting metastatic status based on the proportion of cancer‐positive biopsy cores exhibiting a cribriform pattern. The area under the curve (AUC) was 0.704, indicating moderate discriminative ability. The optimal cut‐off value, determined using the Youden Index, was 0.445, yielding a sensitivity of 57.1% and a specificity of 75.6%.

## Discussion

In our previous study, we demonstrated that the presence of a cribriform pattern and IDC‐P in biopsy specimens was independently associated with lymph‐node metastasis, regardless of the number of involved cores or the percentage of the tumor involved [4]. The study focused on binary assessment – namely, whether these features were present or absent – and used lymph‐node status at prostatectomy as the clinical endpoint (N‐stage). In designing the present study, we deliberately selected non‐metastatic GG 5 cases rather than a Gleason score–matched cohort, because the metastatic group encompassed Gleason 7–10 cases and was heavily enriched for high‐grade disease. This comparator reduces grade heterogeneity and allowed us to address whether a semiquantitative cribriform index provides prognostic information beyond extreme grade at diagnosis. Building upon those findings, the current study explored whether a semiquantitative evaluation, based on the proportion of cancer‐positive cores, could provide additional prognostic value. This time, the presence of distant metastasis was used as the clinical endpoint. Apparent discrepancies with our previous report warrant clarification. That study evaluated the binary presence of cribriform/IDC‐P in relation to pathological lymph‐node metastasis at prostatectomy (N‐stage endpoint). In contrast, the current study addressed a distinct question: at the time of diagnostic biopsy, we evaluated whether the proportional burden of cribriform involvement across cores distinguishes patients with radiologically evident distant metastasis (M‐stage endpoint) from those without. In this cross‐sectional, high‐grade cohort, binary cribriform was common and showed a univariable association; however, it did not remain independent after adjustment for tumor burden and sampling intensity, whereas the per‐core proportion retained independence. Thus, the two observations may be regarded as complementary rather than contradictory: binary presence signals adverse biology across disease stages, whereas proportional burden refines risk stratification specifically at presentation.

By comparing binary and semiquantitative assessment models in a cohort of patients with treatment‐naïve high‐grade CSPC, we aimed to clarify which method better reflects metastatic potential and how these features might be integrated into routine pathology reporting. Our results demonstrated that the cribriform pattern was significantly associated with metastatic status when evaluated as a proportion of cancer‐positive cores, but not when evaluated in binary form. This suggests that semiquantitative evaluation may capture biological aggressiveness more effectively than binary classification [2]. In addition, our size‐stratified descriptive analysis showed that large cribriform morphology was enriched in the mCSPC cohort, whereas small cribriform morphology was relatively more frequent in the non‐mCSPC (Table 2). We therefore present the size descriptor as complementary information, while the prespecified primary endpoint remained the semiquantitative per‐core cribriform proportion. Previous reports have also described adverse associations of large cribriform – defined either as ≥2× the diameter of adjacent benign glands or as exceeding a fixed diameter threshold – with pathological stage and recurrence [10]. However, recognizing large versus small structures on biopsy may be limited by sampling variability and interobserver disagreement. Accordingly, we present the size descriptor as complementary information, while retaining the semiquantitative per‐core cribriform proportion as our prespecified primary endpoint [6]. This association was reinforced by threshold analysis, which indicated that patients with a cribriform pattern in approximately half of the cancer‐positive cores may merit particular attention. However, the discriminatory performance of this threshold was only moderate (AUC 0.704; sensitivity 57.1%), indicating that the cribriform proportion alone cannot serve as a stand‐alone predictor. A reporting threshold around this value may therefore provide complementary rather than definitive information for stratifying metastatic risk, and its interpretation should be contextualized with respect to sampling variability and diagnostic burden. Accordingly, reporting the overall proportion of the cribriform pattern across biopsy cores may enhance prognostic accuracy; however, validation in larger cohorts and integration with other parameters will be essential before clinical application. From a clinical perspective, even within high‐grade disease, a higher proportional burden of cribriform involvement (approximately one‐half of cancer‐positive cores in our cohort) may help flag patients who warrant treatment intensification within guideline‐based pathways – for example, long‐term androgen‐deprivation therapy with dose‐escalated radiotherapy and, in selected very‐high‐risk settings, the addition of a novel androgen‐receptor pathway inhibitor; or radical prostatectomy with extended lymph‐node dissection with adjuvant/salvage strategies determined by the multidisciplinary team [1, 14]. In this study, although IDC‐P was more frequently observed in the mCSPC group and was associated with metastasis in univariable analysis, it did not remain an independent predictor in either the binary or semiquantitative multivariable models. This finding warrants careful interpretation, as IDC‐P is generally recognized as an adverse prognostic feature [3, 11]. The limitations of H&E staining alone may have contributed to under‐recognition, raising the question of whether basal cell markers should be routinely used for confirmation in selected settings. Accordingly, our H&E‐based classification should be regarded as pragmatic but not fully reliable, and potential misclassification may have attenuated associations. Indeed, several prior studies have combined cribriform and IDC‐P into a single composite variable, which may be more practical in routine diagnostics. Although our prespecified endpoint remained the cribriform proportion, future studies should evaluate composite indices incorporating IDC‐P. In addition, we did not separately model the proportions of general Gleason patterns 4 and 5, and therefore cannot determine from the present data whether the cribriform proportion provides independent prognostic information beyond these general patterns. This remains an important question for future validation studies. Additional unresolved issues include whether IDC‐P should be evaluated in a binary or proportional manner and whether its assessment is appropriate in prostate biopsy specimens at all, given the inherent constraints of limited sampling and interobserver variability. These questions remain unanswered in the current study, and further investigation in larger, standardized cohorts is warranted to clarify the diagnostic and prognostic relevance of IDC‐P in prostate biopsy specimens.

Other parameters demonstrated notable associations with metastatic status. The cancer‐positive core ratio was a significant predictor in both models, supporting its utility as a proxy for tumor burden. Although the GG 4 + 5 composition ratio did not remain significant in multivariable analysis, it showed significant differences in univariable comparisons and may still contribute to broader prognostic frameworks. Although all non‐mCSPC cases were GG 5 tumors, the GG 4 + 5 and GG 5 composition ratios were higher in the mCSPC cohort because those patients typically had fewer cancer‐positive cores overall, which inflated the relative proportion of GG 4 + 5 or GG 5 cores. These ratios therefore reflect sampling intensity and tumor distribution rather than an absolute predominance of GG 5 disease, and they did not retain independence in multivariable models. The number of biopsy cores was inversely associated with metastatic status in both models. Importantly, all cores obtained per patient were analyzed; thus, the inverse association with the number of biopsy cores likely reflects clinical sampling decisions in patients with very high PSA rather than analytic subsampling by the study team. Although this approach may be sufficient for diagnostic confirmation, it may limit detailed morphological and molecular assessments. Given the increasing reliance on genomic profiling in prostate carcinoma, particularly in advanced‐stage disease, obtaining an adequate number of biopsy cores, when clinically feasible, should be encouraged. This study had some limitations, including its retrospective, single‐center design, reliance on conventional staging imaging rather than uniform PSMA PET/CT, and absence of interobserver reproducibility metrics. Information on biopsy guidance (systematic versus MRI‐targeted) was not uniformly available, precluding formal comparison between groups and potentially introducing detection bias. Subsequent development of metastasis during follow‐up was not captured because the comparator cohort was defined at diagnosis only. Prospective, multi‐institutional validation is required before adoption into routine practice [5].

Altogether, our findings underscore the potential value of semiquantitative, core‐based evaluation of cribriform pattern for metastatic risk stratification in treatment‐naïve prostate carcinoma. Although the adoption of such reporting may require additional effort, defining practical thresholds – such as involvement of approximately half of the cancer‐positive biopsy cores – may facilitate standardized interpretation. At a minimum, necrosis could be documented in binary form, and the diagnostic role of IDC‐P in biopsies requires further clarification. Future studies integrating histological, immunohistochemical, imaging, and molecular features will be essential for the development of reliable pathology reporting systems that support precision oncology [7].

## Author contributions statement

YO collected the required data from the database, re‐evaluated the specimens, constructed and integrated the dataset and wrote the manuscript. SS assessed histological findings in collaboration with CH and contributed to diagnostic interpretation. RK assisted with case validation and integration of clinical and radiologic information. YY provided radiologic input and supported clinical correlation. SY coordinated specimen access and logistical arrangements. CH reviewed histological slides and discussed key morphologic features with SS. EY participated in specimen review and quality control. KW contributed to diagnostic assessment and internal data validation. RM supported data integration and case tracking within the pathology database. YH contributed to clinical data alignment and reviewed the manuscript. AS and TA provided expert opinions from a urologic perspective and contributed to the clinical interpretation of findings. NN, as a senior urologist, reviewed the findings from a clinical perspective and advised on manuscript revision. HN supported statistical analysis and advised on data interpretation. TK critically revised the manuscript from a urologic standpoint as department head. TY and YM supervised the histological evaluation and substantially revised the manuscript as senior pathologists. All authors reviewed and approved the final version of the manuscript.

## Supporting information


**Table S1.** Composite cribriform–necrosis categories and metastatic status (exploratory analysis)

## Data Availability

The data that support the findings of this study are available from the corresponding author upon reasonable request.
