# Supplementary material for: Cribriform pattern and IDC‐P in prostate biopsies: prognostic relevance and reporting in metastatic disease
Source: J Pathol Clin Res. 2025 Oct 8;11(6):e70052. doi: 10.1002/2056-4538.70052 (PMC12508520; doi:10.1002/2056-4538.70052)
Supplement: Supplementary file 1 — Table S1. Composite cribriform–necrosis categories and metastatic status (exploratory analysis) [file CJP2-11-e70052-s001.pdf]

Cribriform pattern and IDC-P in prostate biopsies: prognostic relevance and reporting in metastatic disease

Y Okubo *et al.* *J Pathol Clin Res* https://doi.org/10.1002/2056-4538.70052

Table S1. Composite cribriform–necrosis categories and metastatic status (exploratory analysis)

Category	Non-mCSPC, *n* (%)	mCSPC, *n* (%)	Total, *n* (%)	OR (95% CI)	*p*
Neither	32 (61.5%)	20 (38.5%)	52 (100%)	Ref	–
Either	37 (39.4%)	57 (60.6%)	94 (100%)	2.47 (1.23–4.94)	0.011
Both	9 (24.3%)	28 (75.7%)	37 (100%)	4.98 (1.95–12.7)	<0.001

Values are *n* (row %). Composite categories were defined as follows: Neither, no cribriform morphology and no tumor necrosis; Either alone, cribriform morphology or tumor necrosis present, but not both; Both, both cribriform morphology and tumor necrosis present. ORs are from univariable binary logistic regression with Neither as the reference (Ref) category; 95% CIs are shown. Overall association was tested by Pearson's $\chi^2 = 13.1$, df = 2, $p = 0.001$; ordinal trend by linear-by-linear association $\chi^2 = 12.8$, $p < 0.001$. *p* values are two-sided. Percentages may not total 100% due to rounding.

CI, confidence interval; mCSPC, metastatic castration-sensitive prostate carcinoma; non-mCSPC, non-metastatic castration-sensitive prostate carcinoma; OR, odds ratio.
